# Cough Test during Tension-Free Vaginal Tape Procedure in Preventing Postoperative Urinary Retention

**DOI:** 10.1155/2013/797854

**Published:** 2013-04-09

**Authors:** Jian Kang, Xin Gou, Qing-hua Zhao, Wei-yang He, Ming-zhao Xiao, Ming Wang, Yuan-zhong Deng

**Affiliations:** Department of Urology, The First Affiliated Hospital of Chongqing Medical University, No. 1 Youyi Road, Yuzhong District, Chongqing 400016, China

## Abstract

*Objective*. To discuss the practical value of the cough test during the tension-free vaginal tape (TVT) procedure. *Methods*. In the first group, 41 patients of female stress incontinence received TVT operations which were performed according to the Ulmsten's method strictly, only that the stress of tape was adjusted in light of the cough test. In the second group, 44 patients of female stress incontinence received TVT operations in which the tape was put under the urethral tract without stress, not adjusted by cough test. *Results*. The cure rate was 38/41 (92.6%) in the cough test group and 41/44 (93.1%) in the noncough test group; detrusor pressure-uroflow study indicated that there were 11 cases in the obstruction zone in the cough test group while only 3 cases were in the obstruction zone in the noncough test group; 4 cases of urinary retention and 5 cases of voiding dysfunction were found in the cough test group, while difficulties of urination were not found in the non-cough test group. *Conclusion*. Adjusting the tape stress in accordance with the cough test during the TVT can increase the opportunity of urinary retention or difficulty of urination after operation. So there is no benefit of the cough test during tension-free vaginal tape procedure in preventing post-operative urinary retention.

## 1. Introduction

DeLancey [1] proposed a theory to explain the mechanistic basis of female stress incontinence in 1994. In his view, the increase in the abdominal pressure during cough in healthy females led to elevated urethral closure pressure and prevented leakage from the urethra. Compression of urethra on its underlying hammock-like supportive tissues was the main reason for the increase of urethral closure pressure; therefore, focus should be placed upon reconstruction of the supportive tissues under the urethra in the treatment of female stress urinary incontinence. On the basis of this theory, Ulmsten et al. [2] firstly designed and reported a method of using tension-free vaginal tape (TVT) procedure for the treatment of female stress urinary incontinence. Because of its minimal injury, short hospital stay, and high cure rate, this procedure has been widely adopted. According to the latest statistics, approximately 68% of female patients with stress incontinence were treated by this operation [3].

Urinary retention and dysuria are the most common complications after TVT. The rate of voiding dysfunction following the TVT procedure has been reported to be between 4% and 10%, and that is between 2% and 43% for urinary retention. Tape adjustment during TVT procedure may influence the rate of urinary retention and the postoperative urinary retention is often related to the amount of tension placed on the sling [4–6]. Less tension without leaving the patient incontinence would be most desirable. To reach the balance of minimizing retention rate while optimizing continence, Ulmsten recommended the use of a cough test intraoperatively. However, it has been difficult to quantify the cough test and to use the results to predict the effect on the continence. Takacs and Medina [7] established a criterion whether each cough would produce a spurt of urine or not to quantify the cough test. Nevertheless, it is hard to quantify the amount of urine, and consequently it has a poor reproducibility in the clinical practice. Even so, Murphy et al. [8] demonstrated that the use of the cough test is associated with greater improvements in stress incontinence. However, Low et al. [9] compared a group of women undergoing a TVT procedure who had a cough test intraoperatively with a group who did not have a cough test and demonstrated no difference in continence postoperatively between these two groups. They questioned the necessity of an intraoperative cough test. In the present study, we also compared one group of women undergoing a TVT procedure with a cough test intra-operatively to the other group who did not have a cough test. We, too, failed to see the benefit of the intra-operative cough test in reducing the rate of post-operative urinary retention and dysuria.

## 2. Material and Methods

In a retrospective analysis, from January 2001 to January 2008, 125 women diagnosed with genuine stress incontinence underwent TVT procedure. Patients with urinary tract infection, bladder and vaginal prolapse, urge incontinence, detrusor instability, low detrusor contractility, previous surgery for SUI, or a maximum flow rate less than 15 mL/s were excluded from this study. In order to exclude the surgeons learning curves impact, the first 20 cases of each group were not included in this analysis. All patients underwent history and physical examination, urine dipstick and culture, and preoperative urodynamic examination, including urethral pressure profilometry, urethrocystometry, uroflometry, and abdominal leak point pressure determination. Patient characteristics, preoperative clinical and urodynamic data are summarized in Table 1. All surgeries were performed under spinal anesthesia. The operation was carried out according to the method described in detail by Ulmsten et al. [2]. Sling tension was intraoperatively adjusted according to the results of the cough test in 41 cases. In particular, following placement of the sling under the urethra, 250 mL of normal saline was infused into the bladder, and then the patient was asked to cough vigorously. When leakage occurred, the sling was tightened gradually until obtaining 2-3 episodes of leakage. In another 44 cases, the tape was tightened so that a pair of scissors could be easily admitted between the tape and the midurethra. The catheter was removed 48 hours postoperatively. Follow-up evaluation was carried out at 12 months after operation. Our criterion of a cure was that a patient remained completely dry during the cough test. If she experienced any leakage, the patient was classified as a failure. All patients underwent urodynamic examination. Based on the results of detrusor pressure-uroflow study, we determined whether there was bladder outlet obstruction. According to the nomogram of Blaivas, we classified any pair of values of free Qmax (maximal flow) and pdet.max (maximal detrusor pressure during uroflometry) into unobstructed zone or obstructed zone (mildly, moderately, and severely). Urinary retention was defined as (1) need for prolonged catheterization or intermittent self-catheterization (2) need for urethral dilation postoperatively. Voiding dysfunction was defined as maximal flow rate (Qmax) of <15 mL/s.

Comparisons between the cough test group and noncough test group were performed with parametric analysis. The independent student's *t*-test was used to compare distributed variables; categorical data were analyzed by the *χ* ^2^ test, a *P* < 0.01 was considered statistically significant. Analyses were performed using a statistical software (SPSS 13.0, SPSS, Chicago, IL, USA).

## 3. Result

Patients' age, menopause status, concurrent chronic illness, previous hysterectomy, and preoperative urodynamic parameters (Qmax, MUCP, and ALPP) were comparable between the cough test group and the noncough test group as presented in Table 1. The efficacy of the procedure was also comparable for both groups. The cure rate was 38/41 (92.6%) in the cough test group and 41/44 (93.1%) in the noncough test group (Table 2). With respect to the bladder outlet obstruction, urinary retention occurred in 4 cases after removal of the urethral catheter in the cough test group, and none in the noncough test group (Table 2). There was a difference between the two groups for postoperative urodynamic parameters concerning bladder outlet obstruction (Table 3). Detrusor pressure-uroflow study indicated that there were 11 cases in the obstruction zone in the cough test group with only 3 cases in the obstruction zone in the noncough test group (*P* < 0.01). MUCP of the cough test group was higher than the noncough test group postoperatively (60.33 + 9.21 cm H_2_O versus 58.62 + 7.83 cm H_2_O). However, there is no statistical significance between the two groups (*P* > 0.01).

## 4. Discussion

The Cough test has been an advocate in determining the correct tension of the tape although it has been very difficult to judge the differences in cough efforts between patients. Murphy et al. [8] demonstrated that the use of the cough test is associated with greater improvements in stress incontinence. Lavy et al. [10] compared a group of women undergoing a TVT procedure who had a cough test intraoperatively with a group who did not have a cough test and demonstrated no difference in cure rate between these two groups. In the study reported by Adamiak et al. [8, 11] patients in the intravenous anesthesia group did not use the cough test and patients in the epidural anesthesia group underwent the cough test during TVT procedure. Again, no significant difference in efficacy was noted between the two groups. Our results from 12 months of postoperative followup revealed that the cure rates achieved were 92.6% and 93.1% in the cough test group and the noncough test group, respectively. This finding indicates that the use of an intraoperative cough test did not generate an observable effect on continence in patients who received TVT procedure.

The same as with other SUI procedures, urinary retention and voiding dysfunction are the most common complications after TVT. Advanced age, concurrent with other gynecological procedures, preoperative low urine flow rate, and reduced bladder contractility are the main risk factors for postoperative urinary retention [12, 13]. In addition to preoperative risk factors, voiding disorder after TVT largely results from excessive tension of the sling placed intraoperatively. Among the 41 cases in which the cough test was used to adjust sling tension, there were 4 cases of urinary retention and 5 cases of voiding dysfunction after removal of the urethral catheter. In the detrusor pressure-uroflow study, there were 11 cases in the obstruction zone. In contrast, no urinary distention occurred in the noncough test group, and only 3 cases were in the obstruction zone demonstrated by the detrusor pressure-uroflow study. Regarding the MUCP, studies have found controversial results. Koelle et al. [14] observed a decrease in the MUCP without statistical significance. Wang [15] observed significantly lower values in the MUCP. In the present study, the cough test group had higher MUCP than the noncough test group postoperatively. But there is no statistical significance. Therefore we concluded that the use of the cough test during the TVT procedure has no benefit in improving the efficacy; on the contrary, the use of the cough test tended to cause excessive sling tension and elevated urethral resistance, thereby may be increasing the incidences of urinary retention and voiding dysfunction. 

## Figures and Tables

**Table 1:** Patient demographics and preoperative urodynamic parameters.

	Cough test group	Noncough test group (control)	*P* value
Mean age (years)	62.24 ± 11.5	63.31 ± 10.36	*P* = 0.622
Postmenopause	32 (78.05)	39 (88.64)	
Concurrent chronic illness	21 (51.22)	27 (61.36)	
Previous hysterectomy	2 (4.88)	4 (9.09)	
Qmax (mL/s)	26 ± 10.02	27 ± 9.12	*P* = 0.206
MUCP (cm H_2_O)	58.43 ± 9.03	57.89 ± 7.54	*P* = 0.451
ALPP (cm H_2_O)	61.25 ± 11.27	62.01 ± 10.84	*P* = 0.091

**Table 2:** Outcome of TVT in cough and noncough groups.

	Cough test group	Noncough test group (control)
	(*n* = 41)	(*n* = 44)
Cure	38 (92.6%)	41 (93.1%)
Failure	3 (7.4%)	3 (7.4%)
Urinary retention	4 (9.8%)	0

*P* > 0.25.

**Table 3:** Postoperative urodynamic parameters.

	Cough test group (*n* = 41)	Noncough test group (control) (*n* = 44)	*P* value
Qmax (mL/s)	27 ± 11.63	26 ± 10.51	NS
Voiding dysfunction	5 (12.2%)	1 (2.3%)	<0.01
Detrusor pressure-uroflow study			
No obstruction zone	30 (73.2%)	41 (93.2%)	
Obstruction zone	11 (26.8%)	3 (6.8%)	<0.01
MUCP (cm H_2_O)	60.33 ± 9.21	58.62 ± 7.83	NS
